# The mutuality account of parenthood: a subjective approach to parent-child relationships

**DOI:** 10.1007/s40592-024-00198-y

**Published:** 2024-07-11

**Authors:** Isabella Holmes, Rosalind McDougall

**Affiliations:** 1 School of Historical and Philosophical Studies, The University of Melbourne, Parkville, Australia; 2 Sydney Health Ethics, The University of Sydney, Sydney, Australia; 3 Melbourne School of Population and Global Health, The University of Melbourne, Parkville, Australia

**Keywords:** Genetic kinship, Genetic relatedness, Ontological security, Parent-child relationship, Parenthood

## Abstract

Stimulated by development of reproductive technologies, many current bioethical accounts of parenthood focus on defining parenthood at or around birth. They tend to exclude from their scope some parent-child relationships that develop later in a child’s life. In reality, a parent-child relationship can emerge or dissolve over time: the parents of person A as an adolescent or adult may be different to her parents when she is a young child. To address this aspect of parenthood, we propose a new ‘mutuality account’ of parenthood, grounded in the concept of ontological security. We argue that in most cases a parent-child relationship exists if there is mutual ontological security between the parent and child. We suggest that this mutual ontological security is constituted and sustained by shared frameworks of reality and cohesive personal narratives. Our intention is to broaden the conceptual understanding of parenthood, to include parent-child relationships that do not fall neatly into current bioethical accounts, and to argue against the notion that objective physiological, causal, or social ties are necessary to ‘make’ a parent.

## Introduction

As the use of new reproductive technologies becomes more common, and social attitudes change regarding blended families, open adoptions, LGBTQI + parenthood and group parenthood, there are more and more parents with ties to their child that look very different to parent-child relationships in the past. Responding to these changes, bioethical accounts of parenthood have often focused on ensuring that the child’s parents are defined at or around birth. Current bioethical literature is often characterised by accounts of parenthood that emphasise the importance of either physiological connections between parent and child, or decisions or intentions involving the child before the child was born.

However, a satisfactory answer to the question ‘What makes a parent?’ needs to also encompass parent-child relationships that develop later in a child’s life. In some cases, a parent-child relationship can emerge or dissolve over time: the parents of person A as an adolescent or adult may be different to her parents when she is a young child. Consider the following case:

A young girl, Mary, has been raised almost solely by her mother, and has court-mandated visits with her genetic father. When she is seven years old, her mother marries a new partner, Ken. Ken has no children of his own and is happy to move in with Mary and her mother, but doesn’t see this as a decision to parent Mary. The three of them move into the same house and set about life together. Mary begins to voluntarily refer to Ken as ‘Dad’. When she is a teenager, Mary decides to change her surname to match her mother’s, and looks into the process of having Ken legally adopt her as his daughter. When she approaches Ken about the decision, he is supportive. Because by law she would need permission from both genetic parents (and her genetic father will not grant this) Mary waits until her eighteenth birthday. She finds that while it is relatively easy to change her name, the adoption of an adult is an arduous process. Mary and Ken decide together that adoption is not necessary because they feel secure in their knowledge that she is his daughter, and that he is her father. Mary ceases contact with her genetic father. While she is not genetically or legally Ken’s daughter, Mary considers Ken her sole father, and Ken considers her to be his daughter.^1^

We suggest that an adequate understanding of parenthood would recognise the relationship between Ken and Mary as a parent-child relationship. While existing bioethical accounts grounded in objective physiological or causal ties between parent and child are useful in most contexts, there is increasing need for additional new ways of defining parenthood that capture relationships like Mary and Ken’s.

Relationships like Mary and Ken’s suggest that the concept of parenthood is bigger than the focus of existing bioethical accounts of parenthood: physiological connection (either genetic or gestational), agreements made before birth, or custodial care. In response to this challenge, we propose a ‘mutuality account’ of parenthood, drawing on the concepts of ontological security and personal narratives. We argue that a parent-child relationship exists if there is a shared ontological security between a parent and child. We suggest that this mutual ontological security is constituted and sustained by shared frameworks of reality and cohesive personal narratives. This approach is a substantial shift in bioethical approaches to parenthood, as it privileges the subjective experience and values of the parent and child, rather than ascribing parenthood based on particular objective features of the relationship.

We have laid out our argument in four sections. In Section I we give an overview of the common approaches to parenthood taken so far in bioethics, demonstrating their limited ability to capture all kinds of parent-child relationships, due to their ascription of necessary physiological features or causal and social roles, or their narrow temporal focus around the birth of a child. In Section II we present two concepts that help build a new, broader account of parenthood. The first, *ontological security*, refers to the background knowledge that we are operating in the same shared reality as those we interact with. This security allows us to function and communicate in our social world. The second, *personal narratives*, refers to the foreground knowledge and stories of our lives, as told by us. This helps us find meaning in our lives, actions, and close interpersonal relationships. In Section III we use these concepts in the context of parenthood, putting forward the mutuality account of parenthood. We argue that a parent-child relationship can exist if there is mutual ontological security, constituted and sustained by cohesive personal narratives between parent and child. This helps to expand the bioethical conceptualisation of parenthood to encompass parent-child relationships which are dynamic, rather than just those ascribed at birth or defined by particular roles. In Section IV we present a complex custody case recently heard in the High Court of Australia, analysing it through the lens of the mutuality account. We demonstrate how this new, subjective account can often better conceptualise parent-child relationships overall, and also assist in understanding and answering complex questions that arise through disputed parenthood.

### Accounts of parenthood in bioethics

Conceptually the term ‘parent’ has been treated in the bioethical literature as a role to be determined when the child is very young, usually by fulfilling particular objective criteria. In the case of accounts grounded in shared genetics or gestation, it is a particular physiological connection, and in the case of accounts grounded in intention to parent, it is the decisions and agreements that have been made before the child has developed their own autonomy. There are also accounts which present the criteria for parenthood as social, with a focus on particular social actions and caring responsibilities as determinative of parenthood.

Broadly, we have determined three approaches to parenthood in the bioethical literature of the last few decades: genetic accounts, gestational accounts, and intentional accounts. It is important to note that these accounts vary widely; some writers argue that their proposed essential feature for parenthood (for example, direct genetic relation) is both necessary and sufficient for the ascription of parenthood, while others posit their proposed feature as merely sufficient. (Bayne and Kolers 2003). (We wish to note that the overview we present is somewhat selective, focused on accounts commonly presented in bioethics journals. We acknowledge that there are many interdisciplinary studies which explore questions surrounding the rights and responsibilities of parents and children. For example, legal literature exploring custodial rights, and sociological literature focusing on the nuanced dynamics between parents and children in our society.)

Genetic accounts hold that the essential feature that grounds parenthood is direct genetic relation. Genetic accounts often align with our social norms and statistics surrounding parenthood (the majority of parents in the world *are* directly genetically related to their children). (Faddy et al. 2018) Genetic accounts of parenthood vary. Strong argues that there are many reasons why parents may value having genetically related children, but that only several are ethically defensible. (Strong 1997) Velleman goes further; when arguing against the ethical permissibility of gamete donation, he claims that it is a moral wrong to create a child who will not have access to direct contact with their genetic mother and father. (Velleman 2005)

Gestational accounts hold that the essential feature for parenthood lies in the process of gestation and childbirth. The basis of these claims is often the physical and psychological labour of the gestating woman, stemming from feminist theories of bodily autonomy and critiques of gestational surrogacy. (Tong 1990) Katz Rothman argues for a gestational account of parenthood by claiming that the connection between foetus and gestating woman has intrinsic value, and that sharing this bond is the only way to have a child of one’s own, from birth. According to Katz Rothman, this calls into question the claim of even genetically related fathers to parenthood from birth. (Katz Rothman 1989) Tong argues for a gestational account of parenthood in response to cases of rejected children born via gestational surrogacy contracts. Tong argues against the practice of commercial surrogacy, arguing that the gestating woman has a stronger claim to parenthood than those commissioning the pregnancy. (Tong 1990)

Intentional accounts hold that the essential feature of parenthood is being involved in some key way in the decisions and actions that help bring a child into existence, or in the decision to be a parent to a particular child. Bioethical accounts that fall under this category vary much more than those encapsulated by genetic or gestational accounts, but for the most part they are utilised in conceptualizing and supporting the practices of surrogacy, adopting and fostering. Haslanger, in response to Velleman’s genetic account, argues for a version of intentional parenthood:

I believe that even in non-kin adoptions where children have no contact with biological relatives, adopted children have families and adoptive parents have children in a sense that is “at least…good enough” and, actually, equal to the relations between biological parents and children. (Haslanger 2009)

While intentional accounts are more subjective and nuanced than genetic and gestational accounts of parenthood, their focus is often on ascribing parenthood through objective caring roles or responsibilities for young children. Other accounts which fall under this ‘intentional’ umbrella include those which define the parent-child relationship as a social one. (Macleod 2018) (Mullin, A. 2014).

Most parent-child relationships (especially when the child is very young) can be captured by a version of these three accounts. We wish to illuminate a previously unexplored aspect of some parent-child relationships– that such relationships are not always consistent over time, and that beyond the fulfillment of essential caring responsibilities which give certain parental rights, there is also a subjective mutual aspect to certain parent-child relationships. A case like Ken and Mary, where Ken has no initial intention to raise Mary as his child, and has no physiological connection to her, and yet is still her parent, suggests that there are parent-child relationships that are dynamic and subjectively defined by the people involved. It is not just the fulfillment of a particular role defined by social norms, but a mutual recognition of being parent and child. These relationships are not always addressed by genetic, gestational, or even intentional accounts of parenthood.

We suggest that often what is meaningful in parent-child relationships, especially as children become adults, is subjective. The psychological bonds that connect parent and child may be grounded in certain physiological factors (for example, both parent and child may value the fact that they come from the same ancestral lineage), or in a narrative of being chosen (an adopted child may feel gratitude and luck for being chosen by their parents), but these bonds are subjective insofar as their value is based in narrative, identity, and belonging. Most bioethical accounts of parenthood have a certain focus, and are very useful for particular questions, but the reliance on particular objective criteria to define parenthood can potentially result in a theoretical account which does not encapsulate two people who self-define as parent and child.

Parenthood is sometimes better understood as a relationship, one that can exist over time if both parent and child recognise the other as fulfilling their role. This is why a subjective account is an important addition to the bioethical conversation about the nature of parenthood, as it can address the changing nature of interpersonal relationships, including the initial asymmetry of the parent-child relationship, and it does not necessitate permanent distinctions between a parent and a non-parent. The concepts we describe below can help build such a subjective account.

### Towards a subjective account: two concepts

#### Ontological security

When a person considers her sense of reality as corresponding with the realities of those she interacts with, she can be described as ontologically secure. This person identifies herself as having a continuous narrative, and because of this background knowledge is able to communicate with other people in the world because they share compatible frameworks of reality. The term has its origins in the writing of existential psychoanalyst Laing. Laing’s project was analysing sufferers of certain kinds of psychoses, claiming that their susceptibility to mental disorder was in part caused by feelings of ontological insecurity. He described having a firm core of ontological security as experiencing our own being as real, alive and whole; an inner consistency having begun at birth and ending with death. (Laing 1960) According to Laing, ontological security was vital to our capacity to handle ordinary circumstances of life, and lacking it creates a perpetual threat to one’s interior existence. (Laing 1960) The term was further developed by Giddens, who defined ontological security as a confidence in the continuity of our self-identity and a ‘sense of the reliability of persons and things’. (Giddens, A. 1990, quoted in Bondi 2014)

Laing and Giddens’ formulations of the term are somewhat binary; they both characterise human beings as being either ontologically secure or insecure. Bondi has developed the concept by taking Laing and Giddens’ formulations and applying them to everyday feelings of insecurity, in an attempt to understand ontological security as a continuum we all move along, rather than as a ‘binary distinction that locates and fixes each of us within two discrete categories.’ (Bondi 2014) Central to Bondi’s account is the occupation of space between the interior and exterior, or the self and the other. It is the movement within this space, according to Bondi, which ‘produces feeling, selves, and the worlds we inhabit.’ (Bondi 2014) She stresses that ontological security is not just a comforting psychological certainty, but a dynamic knowledge that imbues our sense of where we are positioned in the world. (Bondi 2014)

Often bioethical accounts of parenthood argue for grounding the value of parent-child relationships in certain physiological, causal, or social ties, implying that these factors inform our sense of self and where we belong. We argue that it is not the objective criteria (for example, our genetic or gestational connections) that ground our identity and belonging but our sense of ontological security. This security can be achieved and maintained in a parent-child relationship, even if that relationship does not fulfil the criteria described in the previous section.

Consider the following case:

Mitchell was raised by his biological parents, who fulfilled socially recognised caring duties and were loving as he grew into an adolescent. However, as he reached young adulthood, he opened up to his parents and told them he was gay. His parents rejected him, and he turned to his Aunt Lily for support. While he still lived under his biological parents’ roof, his relationship with them started to crumble, and his supportive relationship with his Aunt Lily became crucial for fostering his sense of identity and belonging.

As Mitchell reflects later on, he recognises that he has three parents. His biological parents and his Aunt Lily. Lily never had children of her own, and sees Mitchell as her son, but also recognises the existing connection between Mitchell and his biological parents.

In Mitchell and Lily’s case (as in Mary and Ken’s situation described earlier), ontological security does the work of solidifying their parent-child relationships. The parent and child’s mutual sense of ontological security forms the foundation of a new account of parenthood that captures parent-child relationships that have developed when the “child” is older. This allows us to recognise parenthood as being a relationship where each side recognises and affirms the particular subjective role of ‘parent’ or ‘child’ to the other. A relationship between parent and child that has both parties interacting within a shared framework of reality, constituted by cohesive personal narratives, is one where both have a sense of ontological security.

#### Personal narratives

Our personal narratives are our continuous biographical stories. These are the stories of who we are, where we have come from, and where we intend to go, as well as an account of the people and events that have had particular significance to us over the course of our lives. Our personal narratives inform our sense of ontological security, by acting as our individual, subjective view of ourselves existing over time. This grounds our sense of where we are positioned in the world. While ontological security is a kind of background knowledge that allows us to interact with the world around us, personal narratives are a kind of foreground knowledge, in that we can consciously recognise and retell the ongoing stories of our lives, and through this give meaning to those events or people that have influenced or changed our narrative.

Personal narratives have particular importance in the development of parent-child relationships. Our world-views and narratives are often shaped by our interactions with (and the personal narratives of) our parents. Part of the complex characteristics of parent-child relationships is the asymmetry that exists when the child is very young, and this changes as the relationship progresses through time. Ruddick addresses the two-way influence of parents and children on each other’s personal narratives:

Children are born into their parent’s lives, often radically changing those lives before developing lives of their own. Subsequently, the lives of parent and children may remain inextricably intertwined, whether lived apart or under one roof as frail parents come to depend increasingly on their adult children’s care. Our earlier criteria for individuating lives (one life per one social world) does not work well in regard to family lives, but then neither do our usual moral distinctions and principles. (Ruddick 2005)

Ruddick points to the particular moral nature of the parent-child relationship, something that has special complexity owing to the asymmetry of influence over personal narratives, often beginning with the parent’s narrative as dominant, and, when a parent is frail or has passed away, the personal narrative of the adult child becoming dominant.

Lindemann Nelson characterises the influence of our family on our narratives by describing what kinds of special goods families can contain and convey:

Families are significant contexts… in which we can express parts of ourselves which we elsewhere suppress, places where we can know and be known with a sort of particularity that doesn’t often occur elsewhere. (Lindemann Nelson 1992)

Lindemann Nelson’s conceptualisation puts at the forefront the psychological benefits of sharing a family space. To know and be known is an important aspect of both creating a personal narrative, and having it recognised by those to whom we are closest. Lindemann Nelson argues against ‘the natural kind’ view of family; that family is fundamentally grounded in genetic relation, and instead proposes that valuing the genetic relationship you share with your family is just one of many potential values we can share in our family narratives. (Lindemann-Nelson, J. 1992) We suggest that if both the child and parent have personal narratives that are cohesive and help them sustain ontological security, they are much more likely to be able to fulfil the particular subjective roles that their relationship needs.

### The mutuality account of parenthood

Parenthood can be a relationship mutually affirmed by the cohesiveness of two sets of personal narratives. These narratives build a shared reality for the parent and child to exist within, allowing both parties to feel ontologically secure. This gives a foundation for both parties to recognise and self-define their version of ‘parental’ or ‘filial’ roles. The personal narratives need not be identical. Indeed they can be grounded in lots of different kinds of values (e.g. valuing the genetic connection, or the notion of being ‘chosen’ by adoptive parents), but they must be at least cohesive enough to contribute to the shared reality that will ground the parent and child’s sense of ontological security.

The mutuality account is so named because it stresses the mutually built parent-child relationship as important. What ‘makes’ parenthood overall in these cases like Lily’s or Ken’s is the cohesiveness of our subjective personal narratives, rather than objective connections from physiology or causality. This approach is temporally different to previous accounts, analysing parent-child relationships after they have formed. This allows a broader scope of parent-child relationships to be captured by the account. For example, a child may be raised by a single mother, and reach out to their biological father when they become an adult; any connection they form is not a case of ‘gene calling to gene’, but an encounter of two people with separate personal narratives, getting to know each other enough to mutually build a relationship in a shared reality, eventually allowing both to feel ontologically secure as parent and child.

While ontological security is a property of the self, we argue that relationships are properly described as ontologically secure if both parties have this property as a result of sharing the same framework of reality. This is where the self-defining aspect of the mutuality account comes in. If we posit parenthood as requiring fulfilment of a particular objective criteria then we could potentially look at two people who self-define as parent and child (that is, they each recognise the other as their parent or child) but *do not* fulfil the particular objective criteria we have set out, then we have no choice but to make the claim that even though these two people consider themselves parent and child, they in fact, are not. If both parties are autonomous, this would seem an unusual and problematic response.

We emphasise that this mutuality is a sufficient, not a necessary condition for parenthood. Our subjective account is designed to capture parent-child relationships that would be uncaptured based on the previous bioethical literature, particularly those relationships where the “child” is an older child, adolescent or adult. We are working ground up– taking examples of relationships which are mutually recognised as parent-child relationships and attempting to determine what *makes* a parent in these situations - something other than objective criteria involving genetics, gestation, intention or social roles.

We acknowledge that there are limitations to this view, and many questions that warrant further research: can the mutuality account conceptualise parent-child relationships where a parent or child has died? What about relationships where a child has been manipulated by a more powerful adult? And what level of cognitive development is required for the kind of recognition we denote? Our account in its current iteration cannot give a satisfactory answer to these questions. For this reason, we posit the account as a contribution to an ongoing conversation surrounding the importance placed on biological ties between parents and children, one which illuminates mutual ontological security as an important but under-recognised aspect of many parent-child relationships.

### The mutuality account applied to a complex case

So far, we have introduced a new account to broaden the scope of parent-child relationships conceptualised in bioethics. We have explained how the account works, using the concepts of ontological security and personal narratives and the case examples of Ken and Mary, and Mitchell and Lily.To demonstrate how the account can be useful for understanding complex cases and potentially shed light where parenthood is disputed, we now discuss a custody case recently heard in the High Court of Australia. (Masson & Parsons and Ors. High Court of Australia) (Robert and Kelly 2019) This case concerns younger children and does not have the same obvious intuitive pulls as the two cases previously described. Our description of the case is based on publicly accessible documents. We will present the case, and then use the mutuality account as a tool for analysing the parent-child relationships within it.

Over eleven years ago, Susan Parsons^2^sought to become pregnant via sperm donation. An arrangement was made with Robert Masson, a friend of over 25 years, who agreed to donate his sperm and to co-parent (understood as some financial support and some physical care) any resulting children from the arrangement. A child was born, known in the courts as B. Robert and Susan are listed the parents of B on her birth certificate. During this pregnancy, Susan was in the early stages of a relationship with Margaret, who would go on to become her wife, and legal co-parent of her second child, C, who was conceived via anonymous clinical sperm donation. Both B and C, who are now aged 11 and 10, refer to Robert as ‘Dad’. Robert and his partner Greg have a close relationship with both girls, and Robert has helped make decisions surrounding their education, health, and general welfare.

Legal complications arose when Susan and Margaret decided they wished to relocate from New South Wales to New Zealand, the country of Susan’s birth and the place of their marriage in 2015. Robert brought his case to court in 2017, opposing the move on the grounds that as a parent he should have access to regular time with the girls and a chance for ongoing meaningful involvement in their lives. On the first instance, the Judge Cleary of the NSW Family Court found that Robert was indeed the legal parent of B, citing that ‘biology’ was part of the answer, but not wholly determinative, and that the intention and belief of Robert that he would parent B was relevant, but also not determinative. She claimed that Susan and Margaret were not in a de facto relationship at the time of the conception and birth of B, and as a result Robert, not Margaret, was B’s second legal parent. She also made reference to Robert’s ongoing role in B’s life, asserting that he was ‘a parent in the ordinary sense of the word.’

Susan and Margaret appealed to the same court in 2018 on the grounds that the judge in the first instance had erred in naming Robert a legal parent, because she had failed to apply the relevant law. They won this appeal, by force of a section of the state act (Sect. 14(2) of the SOC Act) which states: ‘If a woman (whether married or unmarried) becomes pregnant by means of a fertilization procedure using any sperm obtained from a man who is not her husband, that man is presumed not to be the father of any child born as a result of the pregnancy.’ (Masson & Parsons and Ors High Court of Australia).

Robert appealed to the High Court. Susan and Margaret, as well as wanting relocation, also want B to spend much less time with Robert, for C to spend time with Robert only at their discretion, and for Robert to be restrained from representing himself as the girls’ father. Robert wishes to be recognised as B’s legal parent, via application of federal law, which has no rigid rules designating who is or is not a parent, instead framing custody cases as to be determined on a case-by-case basis.

This challenging case raises many questions. Is Robert Masson the father of B? Is this because of his genetic material or his ongoing role in her life? If the latter, is he also the father of C? What risks are we taking by characterising Robert this way? The judge in this case is presented with a question of custody: who should the girls be raised by and why? The question we propose to answer is more normative: who are the parents of B and C?

We argue that, based on the publicly available information, Robert Masson is the father of not only B, but C as well. This is because both girls’ relationships with Robert fulfil the criteria of the mutuality account. The relationship between both girls and Robert began at birth, meaning their personal narratives were particularly shaped by Robert’s role in their lives. Because both girls refer to him as ‘Dad’, it seems reasonable to assume he is fulfilling the subjective role in the reality they share. Both their personal narratives and their role fulfilment presumably inform Robert, B and C’s sense of ontological security. Perhaps the process of legal scrutiny will change this, but we argue that what makes Robert a parent of B and C is the mutual recognition of each party of the other’s role in their life, grounded in their mutual sense of ontological security. Robert is the parent of B and C because B and C both recognise him as such.

This is not to say that Robert is ‘more’ of a parent to B and C than Susan or Margaret. Both Susan and Margaret are also the parents of B and C, because they both fulfil their subjective roles as mother to the two girls. The girls have three parents (plus Robert’s partner Greg, who is a close adult in their lives). Greg is not a parent because the girls do not refer to him as such, he may fulfil some social roles often assigned to parents, but what is important in the ascription of parenthood is mutual recognition of each other as such, and the ontological security formed by cohesive personal narratives between parent and child.

Of course, the mutuality account is not a clear-cut tool for assigning custody. Custodial arrangements are not the same as parenthood, and these questions are complex and sensitive. The intention of this account is to begin to recognise previously uncaptured aspects of some parent-child relationships, and to place value outside of the objective criteria often used to conceptualise parent-child relationships.

The mutuality account helps in this case by answering in conceptual terms why certain adults in B and C’s life should be considered their parents, and others not. Importantly, it does so not by pointing to objective features, but by assessing the subjective roles fulfilled by parents and children, so the account better matches how the children and parents view their relationship, as compared to other accounts of parenthood put forward in bioethics.

## Conclusion

In this paper we have proposed a subjective ‘mutuality’ account of parenthood, in response to the often narrow focus of current bioethical literature conceptualising parent-child relationships. We wish to illuminate non-objective features of parent-child relationships, especially as children develop foregrounding narratives and become adults themselves. We argue that a parent-child relationship can exist if there is mutual ontological security about that relationship between the parent and child. We suggest that this mutual ontological security is constituted and sustained by shared frameworks of reality and cohesive personal narratives. Overall, our intention was to broaden the conceptual understanding of parenthood, to encapsulate parent-child relationships that do not fall neatly into the categories currently put forward in the bioethical literature. Ensuring that theoretical bioethics attempts to capture parent-child relationships that are experienced as such by those involved is important. Many morally important questions arise in which recognition of a relationship *as a parent-child relationship* is significant; for example, in medical decision-making or in other fundamental life decisions such as where a child or elderly parent should live. There are important practical implications that follow the theoretical task of aligning the concept of parenthood with the real set of relationships that are experienced as parent-child relationships by those involved.

## Footnotes

1 This is an altered and anonymised account of the relationship of a friend of one of the authors, used with permission.

2 Name changed. All people referenced in the case will be referred to by their court pseudonyms.
